# Global Perspective on the Outcomes of Dietary Pattern and Nutrient-Based Interventions for Heart Failure: A Systematic Review of Randomized Controlled Trials

**DOI:** 10.3390/jcm15218430

**Published:** 2026-10-29

**Authors:** Rubee Dev, Jia Yu (Jennifer) Zhang, Kenny T. Lin, Juma Orach

**Affiliations:** 1 Faculty of Applied Science, MacIsaac School of Nursing, University of British Columbia, 5955 University Blvd., MacIsaac Health Building, Vancouver, BC V6T 1Z1, Canada; 2 Centre for Health Services and Policy Research, School of Population and Public Health, University of British Columbia, Vancouver, BC V6T 1Z1, Canada; 3 School of Medicine, University of British Columbia, Vancouver, BC V6T 1Z1, Canada

**Keywords:** dietary intervention, global perspective, heart failure, randomized controlled trials, review

## Abstract

**Background:** Heart failure remains a leading cause of morbidity and mortality worldwide, with a rising prevalence in low- and middle-income countries. Although pharmacologic management is well established, dietary recommendations remain limited and poorly standardized. The objective of this study was to evaluate the impact of dietary interventions on heart failure outcomes, with a focus on global representation and sex stratification. **Methods:** This was a systematic review of randomized controlled trials that assessed the impact of dietary interventions on heart failure outcomes (signs and symptoms, quality of life, mortality, readmissions). Studies were identified through five electronic databases (Ovid MEDLINE, Cochrane Central Register of Controlled Trials, Cumulative Index to Nursing and Allied Health Literature, PsycINFO, and Web of Science). We initially searched for records published in English between 2012 and 2024 and updated our search to 2026 to reflect more recent literature. The risk of bias was assessed using the Joanna Briggs Institute tool for randomized controlled trials. Data were narratively synthesized by sex and World Bank country classification. **Results:** Of 3457 retrieved references, 21 met the inclusion criteria and were included in the synthesis. Sodium and fluid restriction were the most frequently studied dietary interventions (*n* = 9), with mixed findings. While some studies reported improvements in New York Heart Association functional class and quality of life, there was no consistent benefit on Brain Natriuretic Peptide levels, weight, hospital readmissions, emergency visits, or mortality. No randomized controlled trials were conducted in low- and lower-middle-income countries (LMICs), and only three studies conducted sex-stratified analyses. **Conclusions:** Sodium and fluid restriction showed limited and inconsistent evidence of benefits for heart failure outcomes, particularly in acute settings. This review suggests a critical need for high-quality dietary intervention trials in underrepresented populations, including LMICs and women, to inform equitable and effective heart failure management strategies worldwide.

## 1. Background

Heart failure is a rapidly growing public health issue, affecting more than 64 million people worldwide [1,2]. The lifetime risk of heart failure has increased to 24%; i.e., approximately one in four people will develop heart failure in their lifetime [3]. Heart failure is a life-threatening syndrome leading to significant morbidity and mortality, poor functional capacity, and poor quality of life, which create a substantial burden to the healthcare system [2,4]. According to the American College of Cardiology, heart failure is defined as a clinical syndrome involving symptoms and signs resulting from structural and/or functional impairment of ventricular filling or ejection of blood and corroborated by elevated natriuretic peptide levels and/or evidence of pulmonary or systemic congestion [5].

While there are clear guidelines for the medical management of heart failure [6], guidelines for dietary interventions are less robust. Dietary recommendations, particularly those related to sodium and fluid intake, remain a cornerstone of heart failure management. However, recent evidence has questioned the benefits of routine sodium and fluid restriction, leading to increasingly nuanced guideline recommendations. The 2024 Heart Failure Association (HFA) of the European Society of Cardiology (ESC) consensus statement advises limiting salt intake to no more than 5 g/day in patients with heart failure [7]. Sodium restriction can be associated with poor dietary quality, leading to nutritional inadequacy [8]. There is evidence that the Dietary Approaches to Stop Hypertension (DASH) diet—a diet emphasizing fruits, vegetables, low-fat dairy products, whole grains, poultry, fish, and nuts, while reducing saturated fats, red meat, and sweets—restricts sodium without nutritional inadequacy and reduces heart failure incidence and progression [9,10]. Adhering to a Mediterranean diet, which emphasizes high consumption of fruits, vegetables, whole grains, legumes, nuts, and olive oil, with moderate intake of fish and poultry and low consumption of red meat and dairy products, may also reduce the likelihood of developing heart failure [11]. Interventions such as medically tailored meal kits have also been shown to have potential for quality of life improvement in heart failure [12]. This evolving evidence base highlights the need to critically evaluate dietary interventions in heart failure and to better understand whether evidence is representative across diverse populations and settings.

From a pathophysiology standpoint, unhealthy eating habits may result in worsening of congestive symptoms, increased mortality, and increased cardiovascular disease progression [13]. Diets high in sodium and fat can activate the renin–angiotensin–aldosterone system and sympathetic nervous system, leading to vasoconstriction, sodium retention, and fluid overload [14,15]. Saturated fats and refined carbohydrates can lead to lipotoxicity or glucotoxicity in cardiomyocytes, resulting in mitochondrial dysfunction, oxidative stress, and apoptosis [16]. Both promote systemic inflammation, which impairs endothelial function and exacerbates myocardial stiffness, which can lead to higher ventricular filling pressures and congestion [17].

Prior systematic reviews have evaluated dietary interventions in heart failure management [18,19,20,21], although several key gaps remain. While the incidence of mortality from cardiovascular diseases seems to be declining in high-income countries, there remains an escalating prevalence of cardiovascular-related morbidity and mortality worldwide [22]. Patients with heart failure in low- and middle-income countries may present with different baseline characteristics and stages of disease than patients from higher-income countries, and usage of therapies can also differ [23]. Evaluations to determine which dietary interventions work best in different contexts are currently lacking and are needed to appropriately serve global communities. Another aspect of analysis that has continued to be neglected is the efficacy of dietary interventions in the context of sex and gender groups, which differ in their experience of cardiovascular disease and adherence to self-care recommendations [24]. With respect to heart failure specifically, baseline differences have been observed across men and women with heart failure [25,26], with additional differences in symptom experiences and the trajectory of symptom evolution [25]. Hospitalization patterns have also been shown to differ between men and women with heart failure [27], pointing to the need for increased understanding of how dietary interventions may impact men and women with heart failure differently.

This systematic review evaluated the impact of dietary interventions on heart failure outcomes, specifically in terms of *symptom management* and *disease prognosis*. The findings provide evidence on how specific dietary interventions contribute to improved quality of life, reduced hospitalizations, and better overall prognosis in patients with heart failure. Additionally, this study stratified the heart failure outcomes by sex and evaluated the efficacy of dietary interventions in a global context. Findings from this systematic review will contribute to the development of global health policies and guidelines for heart failure management, adding nuance to the role of diet as a component of treatment.

## 2. Methods

We conducted a systematic review following the guidelines established by the Preferred Reporting Items for Systematic Reviews and Meta-Analyses statement [28] and registered our review in the international prospective register of systematic reviews (PROSPERO: CRD42024564120). The completed PRISMA checklist is provided in the Supplementary Materials. The key terms and search strategies used in this review are stated in Supplementary Table S1.

*Eligibility criteria.* We included English-language publications of primary randomized controlled trials of a dietary intervention in patients with heart failure to minimize confounding factors. We evaluated dietary interventions that focused on dietary composition and eating patterns (e.g., sodium reduction, Mediterranean diet, and DASH diet) rather than weight-loss interventions or caloric restriction alone. Studies were excluded if they were solely evaluating a dietary supplement, fluid restriction or calorie restriction without other dietary interventions such as reducing salt intake or following dietary patterns like the Mediterranean diet or the DASH diet. This decision was made because the review focused on dietary composition and dietary pattern interventions, whereas calorie restriction represents a distinct therapeutic approach aimed primarily at inducing weight loss, with separate physiological mechanisms and outcome pathways. Studies using total parenteral nutrition and multimodal (including non-dietary) interventions were also excluded. To identify the most recent literature, we initially included studies published between January 2012 to April 2024 and supplemented this with studies from a search update covering literature published between April 2024 and October 2026. We selected this period to reflect contemporary and emerging evidence, which is more applicable to present-day decision-making.

*Information sources and search strategy.* The search string was developed and refined in consultation with a librarian. We searched five bibliographic databases (Ovid MEDLINE, Cochrane Central Register of Controlled Trials, Cumulative Index to Nursing and Allied Health Literature, PsycINFO, and Web of Science) using our search string to identify randomized dietary interventions tested in patients with heart failure. Medical Sub Headings (MeSH) terms, keywords, and keyword phrases were used for searching the following concepts: dietary interventions, heart failure outcomes, and clinical trials (Supplementary Table S1). For all included studies, we also reviewed the reference lists.

*Selection of studies.* At least two independent reviewers (JYZ, KTL, JO) screened titles and abstracts of all articles retrieved. Each article was screened by at least two reviewers, and conflicts were resolved through discussion. At least two reviewers (JYZ, KTL, and JO) independently screened articles at the full-text level for inclusion. Conflicts were resolved through discussion, and a third reviewer (RD) was consulted for input when required.

*Data extraction.* We hand-extracted data from all included studies into Covidence (Veritas Health Innovation, Melbourne, Australia). The first reviewer (JYZ, JO) extracted the information, with a second reviewer (KTL, RD) confirming details, with discrepancies resolved through discussion. We extracted the country of the study, setting, study enrollment period, randomized controlled trial groups and intervention, baseline group differences, sample size, mean age of participants, proportion of female participants, and study outcomes relevant to signs and symptoms of heart failure, quality of life, or patient outcomes (Table 1). Studies were also assigned a region and country economic status based on current World Bank country classifications by income level data [29].

Risk of bias assessment. We evaluated the risk of bias of included studies using the revised Joanna Briggs Institute (JBI) critical appraisal tool for the assessment of risk of bias for randomized controlled trials (Supplementary Table S2) [51]. The JBI tool comprises 10 items for internal validation and three for statistical conclusion validity. At least two authors independently evaluated all the included studies with the above risk-of-bias tool. Discrepancies were resolved by discussion to reach consensus. The tool itself does not provide suggestions for how to score the results. Responses to the questions of the risk of bias tool were tabulated, with ‘Yes’ earning one point and ‘No’ or ‘Unclear’ earning zero points, for a total out of 13.

Data synthesis. If it had been appropriate to combine data quantitatively and common metrics had been available across studies, we had planned to conduct a meta-analysis. However, given the remarkable heterogeneity in interventions, heart failure subtypes, evaluated outcomes, reported statistics, and the lack of comparable effect measures across studies, we opted for a narrative synthesis of findings guided by the synthesis without meta-analysis framework [52]. We grouped studies into dietary patterns consistent with heart failure care guidelines as interventions [53,54,55]. The synthesis primarily focused on the effectiveness of these interventions across high- versus low-income countries and on sex-stratified outcomes, whenever available. We used tables to summarize the key study characteristics, including the World Bank classification, sample size (and proportion of males and females), interventions, outcomes and risk-of-bias summary. Outcome measures were most commonly reported as group means or medians, mean differences and hazard ratios. Given the heterogeneity in metrics, which included many composite measures and secondary endpoints, we reported the raw estimates from the primary studies, where at least two studies reported the same endpoint. For each intervention and endpoint, we used the sum of a simple vote count to determine the overall direction and level of corroboration of effects. We prioritized overall findings supported by at least two studies and used the risk-of-bias assessments from the JBI tool to assess the certainty of evidence. Consistent with GRADE guidelines [56] for randomized controlled trials, for each intervention and outcome, we prioritized randomization (Q1), allocation concealment (Q2), blinding (for patient-reported outcomes) (Q4-Q6), incomplete data addressed (Q8) and intention-to-treat analysis (Q9). Subsequently, we described the overall direction and certainty of a consistent effect, or whether findings were mixed or uncorroborated. Limitations of the primary studies and synthesis are incorporated into the discussion.

## 3. Results

### 3.1. Study Selection and Characteristics

**Figure 1:**
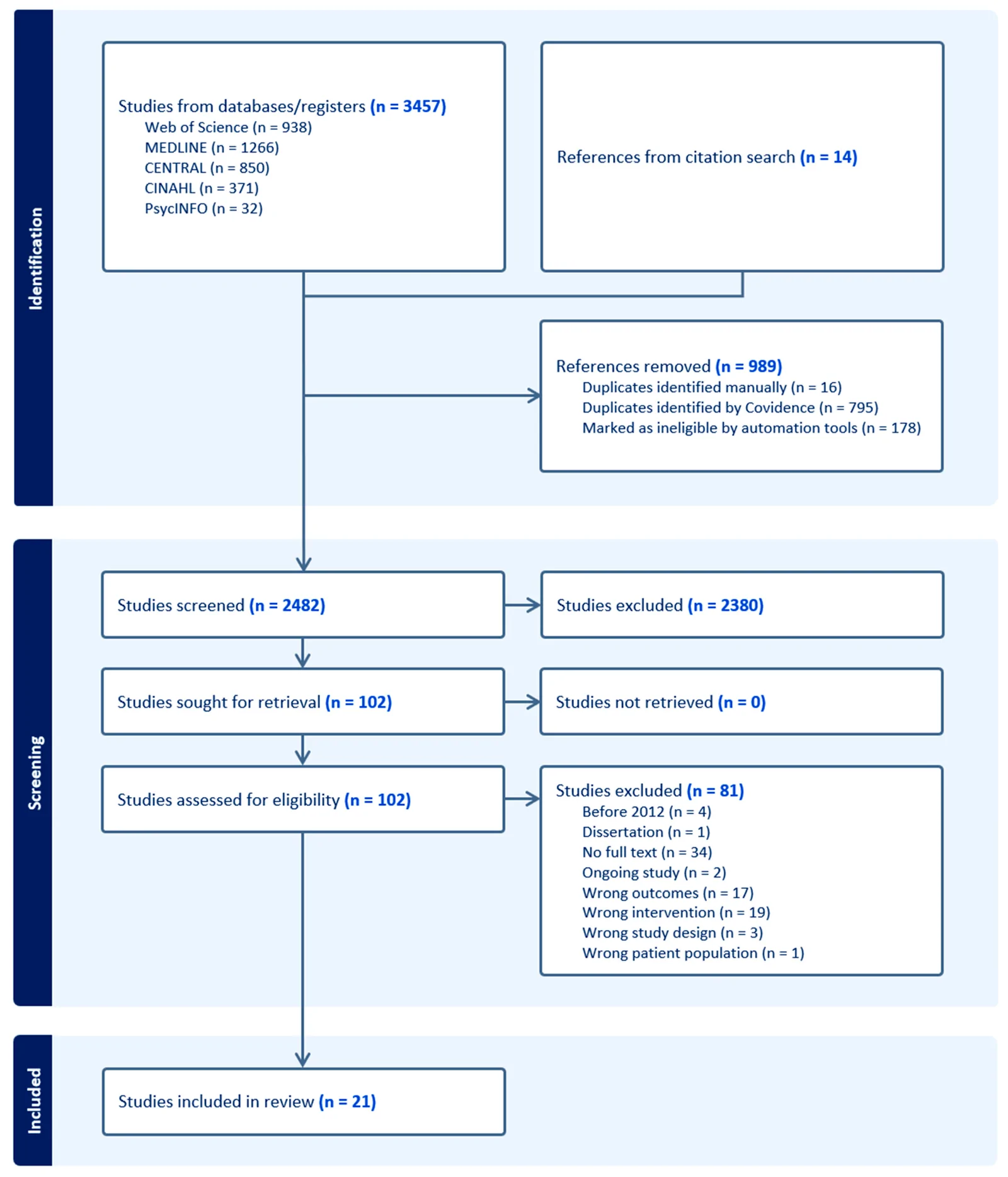
PRISMA flow diagram of search and selection process. The initial search included literature published between January 2012 and April 2024. This was supplemented by a search update using the same MEDLINE (Ovid) approach for literature published between April 2024 and October 2026, in which 370 new results were identified and 5 new studies were added to the final analysis.

Database searches identified a total of 3457 studies. An additional 14 articles were added through citation searching of included studies. Nine hundred and eighty-nine identified in the original search were duplicates or deemed irrelevant by Covidence tools and removed. The remaining 2482 were screened at title/abstract level, with 2380 excluded due to non-relevance. Full texts of the remaining 102 articles were reviewed, and we further excluded 81 studies that did not meet inclusion criteria, including dietary interventions that were solely calorie-restricting [57]. Finally, 21 studies were included in the systematic review [30,31,32,33,34,35,36,37,38,39,40,41,42,43,44,45,46,47,48,49,50] (Figure 1).

**Figure 2:**
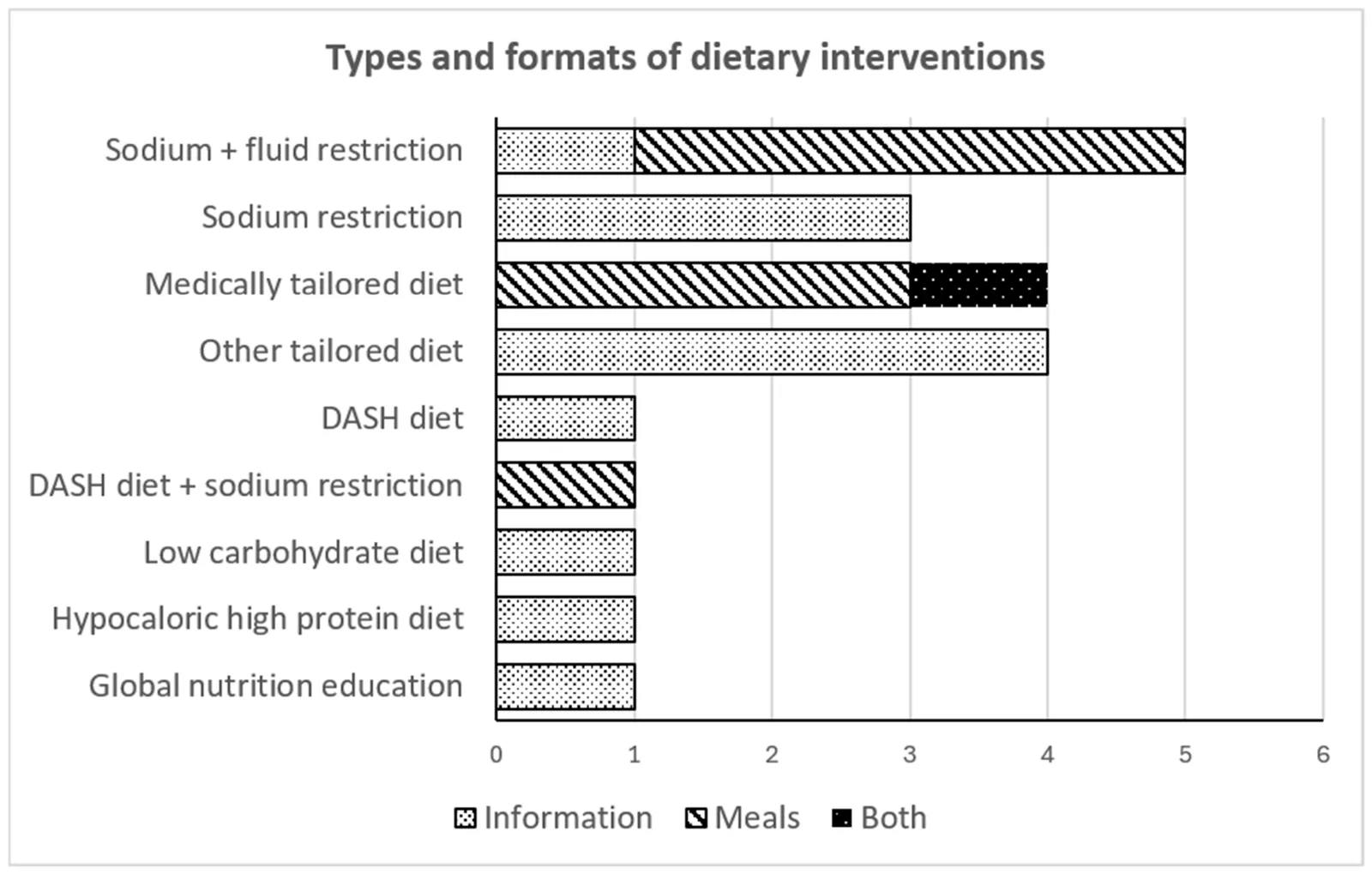
Summary of types and formats of dietary interventions in the included studies. Note: DASH (Dietary Approaches to Stop Hypertension).

Nine (43%) of the included 21 studies utilized a low-sodium dietary intervention, including five where fluid intake was also restricted and another where sodium restriction was combined with a DASH diet [31,36,38,40,41,42,45,46,47]. Four studies used medically tailored meals [30,31,32,37], four used other tailored diet recommendations [34,44,58,59], and one (12%) used only the DASH diet [48]. The remaining three included one each of high protein [39], low carbohydrate [43], and food portion management [34] (Figure 2). Sample sizes varied across studies, ranging from 27 participants to 806 participants [38,40]. The mean age of participants ranged from 57.7 years to 79.2 [39,44]. Three studies used solely hospital-based dietary interventions in the context of acutely decompensated heart failure [42,47,57], while the other 18 interventions were carried out in the community (ambulatory). Heart failure phenotypes were heterogenous. Five studies recruited patients with acute decompensated heart failure, while the rest recruited patients receiving treatment or with a history of heart failure diagnosis. One study recruited only obese patients, and one study recruited only patients with preserved ejection fraction. New York Heart Association (NYHA) class and ejection fraction were inconsistently reported descriptively or as covariates and endpoints.

Ratings of risk of bias for all included studies are shown in Supplementary Table S2, with studies scoring ‘Yes’ on 7–12 out of 13 items on the revised JBI critical appraisal tool for the assessment of risk of bias for randomized controlled trials [51]. Concerns highlighted on risk-of-bias assessment were primarily in domains such as blinding of participants, outcome assessors and those delivering the treatment. For example, most (57%) studies did not mask the intervention from the people delivering it or were unclear.

### 3.2. Results of Dietary Interventions

Sodium Restriction: Overall, the studies evaluating sodium restriction interventions were heterogenous in their specific intervention protocol. Sodium restriction amounts ranged from 0.8 g daily to up to 3 g (Table 1) [42,46,47]. The shortest intervention period was seven days or until the time of discharge [41,42,47] and the longest was 12 months [38]. Interventions ranged from dietitian-led counseling [46], dietary sets of menus and instructions on meal preparation [36,45], allocated salt sachets to add to salt-free meals [41], fully prepped meals shipped to patients’ homes [40], and salt-restricted hospital diet [42,47].

None of the seven studies evaluating Brain Natriuretic Peptide found any statistically significant effect of sodium restriction compared with control interventions [36,40,41,42,45,46].

There was no statistically significant weight loss compared to the control group for sodium restriction dietary interventions [36,40,41,42,47]. Among the three studies that evaluated New York Heart Association (NYHA) functional class [38,42,46], Ezekowitz et al. (2022) (Odds ratio: 0.59, 95% Confidence interval: 0.40–0.86; *p*-value = 0.006)) and Philipson et al. (2013) found the sodium-restricted group had a greater likelihood of improving NYHA class by one compared to the normal care group at 12 months and 12 weeks, respectively [38,46]. Notably, Philipson et al. (2013) also restricted fluid intake, and NYHA class was not a primary endpoint in either study, although it was included in the composite primary endpoint [46]. Additionally, while allocation concealment and participant and diet coordinator blinding were insufficient in both studies, outcome assessors were blinded, and both studies demonstrated rigor in all other items of critical appraisal.

Quality of life was evaluated by the Kansas City Cardiomyopathy Questionnaire (KCCQ) in three studies [38,40,45], the Minnesota Living with Heart Failure Questionnaire in one study [36], and a 36-item Short Form Survey in one study [46]. There was a positive difference in change after 12 weeks in the 1.5 g sodium/day restriction group compared to a control group (14 units: 95% CI: 8–20) in one [40], and a positive mean difference (mean between-group difference: 3.38 points; 95% CI: 0.79–5.96) from baseline to 12 months in the other [38]. Notably, KCCQ was not a primary endpoint, outcome assessors were not blinded in both studies, and participants were not blinded in one study [38]. Moreover, Kalogeropoulos et al. (2020) also restricted fluid intake and did not clarify their randomization methods [40]. No comparative difference in quality of life at six months was noted by Colin-Ramirez et al. (2015) using the KCCQ, although there was an increase in the sodium-restricted group over time [45]. These findings of benefit of sodium restriction were not replicated in studies using other quality of life measures [36,46].

Congestion measured by the clinical congestion score was reported by two studies evaluating sodium restriction in the in-patient setting [42,47]. One study reported the sodium-restricted group to be more congested at 30-day follow-up [47], while the other reported a neutral effect of sodium restriction on the relief of clinical congestion during hospitalization [42]. Reportable symptoms, including general well-being, fatigue, dyspnea, and leg edema, were evaluated by a single study each [41,46]. A dietary intervention using sodium restriction of 2–3 g for 12 weeks reduced leg edema compared to controls [46]. There were no findings for the other three evaluated symptoms [41,46]. No statistically significant improvement in physical limitation score was observed in the 6 min walk test [38].

Sodium restriction was not significantly associated with HF-specific readmissions [36,47], cardiovascular-related hospitalization [38], cardiovascular ED visits [38], all-cause readmissions [42,47], all-cause ED visits [42,47], all-cause mortality [38,42], a composite of mortality and hospitalization [40], or a composite of hospitalization, ED visits or all-cause death [38]. Within an in-hospital sodium-restricted intervention, patients specifically with elevated Brain Natriuretic Peptide >700 pg/mL at 30 days had a higher readmission rate compared with controls [47]. A study by Ezekowitz et al. (2022) in ambulatory care found that dietary sodium reduction (to a target of <1500 mg/day) in patients with heart failure did not reduce cardiovascular-related hospitalization compared with usual care over 12 months [38]. In another 12-week community sodium restriction study, Kalogeropoulos et al. (2020) noted that the Kaplan–Meier event rate of hospitalization at 24 weeks did not differ statistically significantly between arms [40]. One study found mean hospitalization duration to be shorter for normal sodium diet patients compared to sodium-restricted-diet patients [41].

Medically tailored diets: Two studies reported a reduction in 90-day heart-failure-specific hospitalization after medically tailored meal delivery interventions; Eberly et al. (2026) reported a relative risk of 0.29 (95% CI: 0.10–0.87) after 8 weeks of meals based on Navajo recipes [30], while Go et al. (2022) reported an adjusted hazard risk of 0.52 (95% CI: 0.32–0.86) after 10 weeks of meals compliant with Kaiser Permanente Northern California guidelines [37]. Similarly, Pandey et al. (2026) reported an improvement in a win-ratio-based hierarchical composite, including heart failure hospitalization after 12-week meal delivery [31]. All studies had insufficient blinding (or related information) for treatment allocation, participants, and people delivering meals, while Pandey et al. (2026) also had baseline and follow-up differences between and within intervention and control cohorts [31]. Only Pandey et al. (2026) had heart failure hospitalization (readmission) as a primary endpoint, but it was not significantly associated with meals tailored to health condition [31]. Conversely, observed effects of medically tailored meals on quality of life (KCCQ) and body weight, as well as composite scores, including heart failure-specific ED visits and all-cause hospitalization, are mixed, with no clear overall direction [30,32]. Similarly, the potential positive impacts on all-cause mortality, adherence, malnutrition and sarcopenia risk were reported by singular studies that require follow-up by future studies.

Other tailored diets: Personalized dietary recommendations were associated with improvements in 6 min walking distance (m) in elderly patients at 2 months (Mean ± SD = 442.53 ± 54.36 vs. 396.39 ± 48.63, *p* < 0.001) [35], patients with low fiber intake at 12 months (Median (IQR) = 232.50 (214.75, 247.25) vs. 221.50 (203.00, 232.50), *p* = 0.001) [34], and malnourished patients at 12 months (Mean Δ ± SD = −6.93 ± 42.91 vs. 31.30 ± 48.62, *p* = 0.002) [33]. In these populations, personalized dietary recommendations were also associated with improvements in nutritional status assessed using different endpoints: serum markers (albumin, prealbumin and transferrin) [33,34], protein, body fat and body fat percentage [33,34,35]. Study participants and coordinators were not blinded in all three studies, and two of the three studies [34,35] also had potential risk of bias from insufficient investigator blinding, allocation concealment and follow-up reporting. Impacts on Mini Nutritional Assessment scores in malnourished patients were mixed [33,44]. Individualized dietary recommendations were also associated with positive impacts on risk of heart failure hospitalization and all-cause death, gut microbiota, metabolic equivalents and systemic inflammatory biomarkers, but these effects were only identified in singular studies [33,34,35,44]. Impacts on quality of life, anthropometric parameters and all-cause mortality and hospitalization are mixed [33,34,44,59].

DASH: Two studies utilized the DASH diet as the dietary intervention and Brain Natriuretic Peptide level as the outcome, reporting no statistically significant differences between the intervention and control groups [48,50]. Quality of life scores measured by the Minnesota Living with Heart Failure Questionnaire were improved at 3 months in patients provided with DASH diet tools and education compared to the control group [48], but there was no impact on quality of life measured by the KCCQ [50]. Six-minute walk test results were improved in patients following a DASH regimen at three months (124 versus 81, *p*-value = 0.018) in one study [48]. There were no differences observed with respect to hospital readmissions, duration of hospitalization during readmissions, and all-cause death [50].

Global nutrition orientation: A single study evaluating a 1 h global nutritional orientation reported improvements in nutritional knowledge, caloric, fat and sodium intake after 6 months, but no impacts on quality of life or anthropometric parameters [59].

High Protein: A single study evaluating high-protein dietary intervention saw no differences in weight loss, with both high-protein and regular protein menu guides being equally effective [39].

Low Carbohydrate: A single study evaluating a low carbohydrate dietary intervention found no effect of the intervention on weight loss [43].

### 3.3. Comparison by World Bank Income Category

All studies identified by the inclusion criteria were conducted in upper-middle-income countries (eight studies, 44%) [34,35,36,41,42,43,47,49] or high-income countries (twelve studies, 50%) [30,31,32,33,37,39,40,44,45,46,48,50]. One study spanned several countries and included both upper-middle- and high-income countries [38]. There were no studies from low- or lower-middle-income countries. Geographically, two studies were conducted in the East Asia and Pacific region [35], six in Latin America and the Caribbean [36,41,42,43,49], eight in North America [37,39,40,45,50], three in Europe and Central Asia [45,46], and one in the Middle East and North Africa [48]. One study was multinational and spanned regions of North America, East Asia and the Pacific, and Latin America and the Caribbean [38]. Interestingly, all trials conducted in hospital environments were in UMICs. Studies generally did not report on the same variables (Table 1), so there was insufficient data to report on the effectiveness of interventions across different World Bank-categorized economies. Significant impacts of sodium and fluid restriction on NYHA class and KCCQ were reported in studies spanning both HICs and UMICs [38,40,46]. Similarly, the impacts of tailored diets on nutritional and functional status were reported in both HICs and UMICs [33,34,59]. Reductions in 90-day heart failure-specific hospitalizations after medically tailored meal delivery were reported only by studies in HICs [30,37].

### 3.4. Sex-Stratified Data 

The percentage of female participants in the studies ranged from 12% to 62.5%. Only three studies (19%) had females representing half or more of their participants [43,44,45] and used diet optimization, sodium restriction, and low carbohydrate interventions, respectively. Secondary subgroup analyses including sex as an interaction term were conducted in three studies [30,38,44]. An intervention of tailored dietary information was more effective at decreasing all-cause death and heart-failure-related readmissions in females than males, although the authors attributed this to small sample sizes [44]. Ezekowitz et al. (2022), with a multinational community-based sodium restriction intervention [38], and Eberly et al. (2026), with a medically and culturally tailored meal intervention [30], found no statistically significant treatment–sex interactions for quality of life and all-cause hospitalization or ED visits within 90 days, respectively.

## 4. Discussion

This systematic review aimed to assess the impact of dietary interventions on the signs and symptoms, quality of life, and clinical outcomes of heart failure. Overall, sodium and fluid restriction seemed to have an impact on NYHA functional class improvement in patients with heart failure living in the community. However, the benefits on improvement in quality of life were mixed. Furthermore, there was no evidence of any benefit on Brain Natriuretic Peptide reduction, weight loss, hospital readmissions, emergency department visit reduction, or decreased mortality from sodium and fluid restriction. Medically tailored diets may have a positive impact on 90-day heart failure-specific hospitalization, and other tailored diets may improve nutritional status and 6 min walk test results, but the interventions and measures of nutritional status were heterogenous. Other dietary interventions included in this review (DASH diet, high-protein, low-carbohydrate, and global diet education) were represented by two or fewer studies each, with no corroboration of findings, precluding the drawing of definitive conclusions. To our knowledge, this is the first review of dietary interventions in heart failure with a stated intention to evaluate dietary interventions for effectiveness globally and to evaluate sex-stratified analyses. Our review identified a lack of randomized controlled trials evaluating heart failure interventions in low- or lower middle-income economies, illustrating a gap in knowledge regarding interventions that may best benefit these countries. Studies also did not regularly report sex-stratified data, and there remains an underrepresentation of females.

Although some observational studies support the concept that sodium and fluid restriction improves heart failure outcomes [60], the findings of our review show limited beneficial clinical impacts of sodium and fluid restriction in patients with heart failure. Similar findings of a lack of benefit on readmission and mortality were found in prior reviews [19,20,21,61]. Sodium restriction can lead to increased renin–angiotensin–aldosterone system activation, which could trigger the body to retain more water and sodium, potentially worsening congestion [62]. The impact of sodium restriction might also vary by individual; while some may not benefit from it, others might even experience harm [61]. Notably, sodium and fluid are often restricted concurrently; therefore, observed side effects may be related to both or either restriction. Given that uncertainty remains regarding the effect of sodium restriction on heart failure outcomes, further studies on these dietary interventions may be warranted.

Our findings are consistent with the evolving evidence base surrounding dietary sodium restriction in heart failure. A recent overview of systematic reviews found insufficient evidence to support routine dietary sodium restriction for improving mortality, rehospitalization, or quality of life among patients with heart failure and suggested that more stringent sodium restriction (≤2000 mg/day) may even be associated with adverse clinical outcomes in some populations [63]. Furthermore, a 2026 meta-analysis of 16 randomized controlled trials reported that sodium-restricted diets were associated with increased risks of all-cause and cardiac mortality, while providing no significant reduction in heart-failure-related or all-cause readmissions [64]. Although some improvements in quality of life were observed, these findings underscore the ongoing uncertainty regarding the optimal degree of sodium restriction in heart failure management and support the recent shift toward more individualized dietary recommendations. However, our review reveals heterogeneity in available literature on tailored dietary recommendations. More studies, with greater harmonization of methodology, will elucidate the most beneficial tailored dietary patterns.

An important identified gap was the lack of data on dietary interventions in low- and lower-middle-income countries. As a result of ongoing industrialization and globalization, the burden of cardiovascular disease in low- and middle-income countries has increased, accounting for nearly 80% of global cardiovascular disease-related deaths [65]. Heart failure is specifically increasing in low- and lower-middle-income countries [23], and additional evidence suggests increasing mortality among the same [66]. Western strategies imposed on health interventions internationally may not always benefit and can sideline the true needs of other global communities [67]. Therefore, there is an urgent need for evidence on dietary interventions that can best support heart failure patients in low- and middle-income countries.

Similarly, only three studies among the included papers conducted a sex-stratified analysis. Heart failure has been shown to differ in men and women [25,26,27], with a suggestion that some pharmacological interventions may also have greater improvement in women [26]. Consistent with this, tailored dietary information was more effective at decreasing all-cause death and heart failure-related readmissions in females than males in one study [44]. However, sex did not modify the relationship between sodium restriction and medically tailored meal delivery on quality of life or clinical outcomes, respectively, in the other two studies [30,38]. There remains a consistent underrepresentation of women in heart failure trials [68]. Therefore, there remains an ongoing need for increased representation of women in heart failure trials, and to assess how dietary interventions may differentially improve outcomes for women.

The strength of this review was the use of a comprehensive search string in a wide range of database searches. Additionally, investigating the impact of sex and national socioeconomic status globally provides insight into the nature and limitations of the available research. However, this review has some limitations. There was substantial heterogeneity in the intervention protocols, with studies varying widely in terms of sodium targets, intervention durations, heart failure subtypes, delivery methods, and outcome measures, which prevented accurate data pooling and limited the comparability of the results. While we acknowledge that clinical heterogeneity, including differences in heart failure subtypes, may influence the observed trends, a subtype-specific analysis was beyond the scope of this review. The evidence base was also narrow, with most studies focusing primarily on sodium restriction and few evaluating alternative dietary interventions, such as DASH, high-protein, or low-carbohydrate diets, restricting the ability to draw conclusions about the broader role of diet in heart failure management. Furthermore, the studies included in the review did not specify whether background dietary intake was measured or controlled for in the included RCTs. This represents a potential confounder, as individuals who modify one aspect of their diet typically make concurrent changes to other dietary components. Hence, it is important to conduct well-designed, high-quality nutrition interventions that adequately control for or monitor participants’ background diet. Without such controls, findings may be confounded, contributing to mixed results observed across studies. Moreover, although calorie-restriction interventions have demonstrated potential benefits in obesity-related HFpEF, these interventions were outside the scope of the present review. Future systematic reviews specifically examining weight-loss and energy-restriction strategies in heart failure populations are warranted. Methodologically, as the studies were restricted to randomized controlled trials, small sample sizes may have been inadequately powered to detect changes. Moreover, several studies were pilot studies, and many of the outcomes reported here were secondary endpoints that the trials were underpowered to assess. Even when two or more studies corroborated findings, we found risk of bias due to insufficient reporting on concealment of allocation and blinding of participants and staff in many studies, which may disproportionately affect patient-reported measures like KCCQ. Because we focused on recent literature (published after 2012) to reflect contemporary interventions, we could have missed insights from literature published before that. Additionally, no studies have been conducted in low- and lower-middle-income countries, raising concerns about the generalizability of the findings to settings where the heart failure burden is rapidly growing. The underrepresentation of women in studies, combined with limited sex-stratified analyses, limits our understanding of the potential sex-specific effects of dietary interventions.

## 5. Conclusions

This systematic review found that there were no randomized controlled trials evaluating dietary interventions in heart failure in low- and lower-middle-income countries, and very limited sex-stratified data, pointing to an urgent need for evaluation of dietary interventions in these populations. Sodium and fluid restriction, the most studied dietary intervention in heart failure, showed no consistent benefit on key clinical outcomes such as Brain Natriuretic Peptide reduction, weight loss, hospital readmissions, or mortality. While some improvements were noted in the NYHA functional class and potentially quality of life in community-based sodium restriction studies, these findings were not uniform across studies, and there was limited evidence on other symptomatic outcomes. Similarly, conclusions about improvements in 90-day heart-failure-specific hospitalization, nutritional status and 6 min walk test results due to tailored diets are limited by significant methodological heterogeneity. Other dietary interventions (e.g., DASH, high-protein, low-carbohydrate, and global dietary education) have been understudied, limiting definitive conclusions regarding their efficacy in heart failure management. There is also a need for future studies that investigate outcomes by heart failure subtype for more personalized policy and practice applications.

## Figures and Tables

**Table 1:** Characteristics of studies on dietary interventions for heart failure (*n* = 21).

Author/Year	Survey Year	Country	Region	World Bank Class	Sample Size	Age [Mean (SD)]	Intervention Category (Format)	Specific Intervention	Endpoints: *Primary*, Others	Main Findings	Risk of Bias Score
Eberly et al., 2026 [30]	2025	United States	North America	HIC	206; Male = 119 (58%), Female = 87 (42%)	65.8 (14.2)	Culturally and medically tailored meal program (meals)	8-week culturally and medically tailored meal program for patients with a hospital encounter in the preceding 12 months, followed up for 12 weeks	*All-cause hospitalization or ED visit within 90 days*, HF hospitalization, HF ED visit, KCCQ score, BNP, food insecurity, financial strain, blood pressure, body weight	The intervention reduced all-cause and HF-specific hospitalization and ED visits. KCCQ, food security, financial strain, weight and blood pressure were improved in the intervention arm	8
Pandey et al., 2026 [31]	2024–2025	United States	North America	HIC	150; Male = 91 (61%), Female = 39 (39%)	59.5 (52.0–66.0) *	Medically tailored meal delivery (meals)	Medically tailored meal or fresh produce delivery for 12 weeks for patients recruited within 14 days of discharge	*HF readmission or ED visit within 90 days*, win-ratio-based hierarchical composite (all-cause death, total HF hospitalizations or ED visits, and >10-point KCCQ-CSS improvement), KCCQ-CSS	Food supplementation was not associated with an improvement in 90-day readmissions or ED visits. The hierarchical composite and KCCQ-CSS favored food supplementation vs. usual care	7
Compher et al., 2025 [32]	Not stated	United States	North America	HIC	46; Male = 23 (50%), Female = 23 (50%)	67.8 (13.1)	Medically tailored meal delivery (meals)	Crossover medically tailored meal delivery for four weeks after discharge in patients with malnutrition risk, followed up at 30 and 60 days post-discharge	*Malnutrition risk*, 30-day readmissions, ED visits, AHA diet goals, sarcopenia risk	Provision of at least 7 medically tailored meals per week was associated with improved malnutrition and sarcopenia risk and diet adherence	9
Ortiz-Cortés et al., 2025 [33]	2018–2021	Spain	Europe and Central Asia	HIC	64; Male = 43 (67%), Female = 21 (33%)	72.1 (11.2)	Tailored diet (Information)	Personalized dietary plan for patients at risk of malnutrition, followed up at 3 and 12 months	*All-cause mortality or time to first HF hospitalization*, functional capacity (6MWT), nutritional status	Improved functional capacity (6MWT) and nutritional status in the intervention group. No impact on all-cause mortality or time to first hospitalization	10
Li et al., 2025 [34]	2021–2022	China	East Asia and Pacific	UMIC	100; Male = 72 (72%), Female = 28 (28%)	Study group = 66.9 (7.5), Control group = 67.8 (6.7)	Tailored diet (information)	Personalized dietary guidance for patients with fiber intake of <15 g/day, followed up at 1 year	Cardiac function (LVEF, LVEDVI, LVESVI), quality of life (Barthel index and 6 min walking distance), serum nutritional indicators, serum inflammatory markers, plasma TMAO, abundance of gut microbiota	High-fiber diet was associated with increases in quality of life and nutritional indicators and decreases in TMAO and inflammation. No impact on cardiac function	7
Zhou et al., 2023 [35]	2019–2021	China	East Asia and Pacific	UMIC	90; Male = 52 (58%), Female = 38 (42%)	69.3 (10.9)	Tailored diet (information)	1-to-1 consultations regularly with a nutritionist on using hands as food exchange portion estimator for 6 categories of food for 2 months	Nutritional states, 6 min walk test, MetS	Education intervention on food exchange portions improved nutritional states, cardiopulmonary function and exercise endurance in patients with HF	7
Ivey-Miranda et al., 2023 [36]	2018–2019	Mexico	Latin America and the Caribbean	UMIC	70; Male = 47 (67%), Female = 23 (33%)	60.0 (12.0)	Sodium restriction (information)	Nutritionist provided recipes for 2 g sodium restriction, nutritionist provided education, nutritionist visit at 6 and 12 weeks with additional nutritionist advice	*NT-proBNP*, MLHFQ, weight loss, quality of life, hospital readmissions, blood pressure, electrolyte imbalances	Overall poor adherence. No improvement in NT-proBNP among those allocated to sodium restriction (2 g of sodium/day); however, those who achieved Na intake <2500/day had improved NT-proBNP and quality of life without adverse events	12
Go et al., 2022 [37]	2020–2021	United States	North America	HIC	641 with HF (1977 total); Male = 375 (59%), Female = 266 (41%)	71.4 (13.8)	Medically tailored meal delivery with or without nutritional counseling (meals and information)	1 meal/day delivered tailored to KPNC guidelines for heart failure, diabetes and chronic kidney disease for 10 weeks, nutritional counseling	*All-cause hospitalization within 90 days*, All-cause and cause-specific hospitalization or death	Meal delivery did not reduce risk of all-cause hospitalization, but was associated with lower mortality and HF hospitalizations	10
Ezekowitz et al., 2022 [38]	2014–2020	Canada, Australia, New Zealand, Colombia, Mexico, Chile, United States	North America, East Asia and Pacific, Latin America and the Caribbean	UMIC and HIC	806; Male = 538 (67%), Female = 268 (33%)	67.0 (58.0–74.0) *	Sodium restriction (information)	Sample menus for 1.5 g sodium restriction for 12 months, behavioral counseling	*Composite of cardiovascular-related admission to hospital, ED visit or all-cause death within 12 months*, KCCQ, 6 min walk test, NYHA functional class	Dietary intervention to reduce sodium intake did not reduce clinical events in ambulatory patients with heart failure	10
Evangelista et al., 2021 [39]	Not stated	United States	North America	HIC	76; Male = 55 (72%), Female = 21 (28%)	57.7 (9.7)	Calorie restriction with high or standard protein diet (information)	Hypocaloric high-protein diet (30% protein) meal plans for 12 weeks, counseling and nutritional support with licensed dietitian	Weight loss, cardiometabolic risk	Both high- and standard-protein diets reduced weight The high-protein diet resulted in greater reductions in cardiometabolic risks	8
Kalogeropoulos et al., 2020 [40]	2015–2017	United States	North America	HIC	27; Male = 22 (81%), Female = 5 (19%)	62.0 (10.5)	Sodium and fluid restriction (meals)	Prepared tailored meals with either 1.5 g or 3 g daily sodium restriction for 12 weeks	*On-study retention*, KCCQ, BNP, weight loss, composite of mortality and hospitalization	Both control and intervention diets reduced urinary sodium without adverse safety or quality of life signals	10
Fabricio et al., 2019 [41]	2014–2016	Brazil	Latin America and the Caribbean	UMIC	44; Male = 26 (59%), Female = 18 (41%)	57.9 (12.0)	Sodium and fluid restriction (meals)	Low (1.2 g) or normal (2.8 g) sodium content in hospital-prepared meals for 7 days of hospitalization or until discharge	*Serum sodium*, general well-being, dyspnea, BNP, weight loss, hospital readmissions, all-cause death	Low and normal sodium diets achieved similar degrees of decongestion during acute decompensated HF treatment	12
Machado d’Almeida et al., 2018 [42]	2013–2016	Brazil	Latin America and the Caribbean	UMIC	53; Male = 36 (68%), Female = 17 (32%)	72.3 (11.7)	Sodium and fluid restriction (meals)	Restriction to 0.8 g sodium and 0.8 L/d fluid in hospital meals for 7 days or until discharge	*Weight loss*, BNP, clinical congestion score, hospital readmissions, all-cause death	Aggressive sodium and fluid restriction did not provide symptomatic or prognostic benefits and did not seem to have an important neurohormonal effect in patients admitted for decompensated HFpEF.	11
Gonzalez-Islas et al., 2017 [43]	2011–2013	Mexico	Latin America and the Caribbean	UMIC	88; Male = 35 (40%), Female = 53 (60%)	69.4 (12.5)	Low carbohydrate diet (information)	Low (40%) carbohydrate diet for 2 months, nutritional handbook with menus provided	*Oxygen saturation*, body composition, handgrip strength	Low carbohydrate diet improved oxygen saturation but resulted in no significant changes in body composition or handgrip strength in patients with chronic stable heart failure	10
Bonilla-Palomas et al., 2016 [44]	2012	Spain	Europe and Central Asia	HIC	120; Male = 45 (37.5%), Female = 75 (62.5%)	79.2 (7.0)	Tailored diet (information)	Tailored recommendations starting at admission lasting 6 months, nutritional supplements when nutritional goals not reached	*Time to composite of all-cause death or readmission for HF*, time to all-cause mortality, time to readmission for HF	Diet optimization in malnourished hospitalized HF patients reduces risk of all-cause death and risk of readmission for HF	9
Colin-Ramirez et al., 2015 [45]	2012	Canada	North America	HIC	38; Male = 18 (47%), Female = 20 (53%)	65.5 (56.3–72.1)	Sodium restriction (information)	Low (1.5 g) or moderate (2.3 g) daily sodium restriction; patients self-prepare using sample menus for 6 months	*Feasibility*, KCCQ, BNP	Sodium restriction dietary intervention was feasible. While there were no differences between treatment groups, achievement of <1.5 g daily sodium was associated with lower BNP and improved quality of life	10
Philipson et al., 2013 [46]	Not stated	Sweden	Europe and Central Asia	HIC	97; Male = 58 (60%), Female = 39 (40%)	74.9 (8.1)	Sodium and fluid restriction (information)	2–3 g individualized sodium restriction for 12 weeks, coached by dietitian with follow-up every 4 weeks, fluid restriction	*Composite of NYHA functional class, hospitalization, weight, peripheral edema, quality of life, thirst and diuretics*	Individualized salt and fluid restriction can improve signs and symptoms of HF with no negative effects on QoL in patients with moderate to severe HF	10
Aliti et al., 2013 [47]	2009–2012	Brazil	Latin America and the Caribbean	UMIC	75; Male = 52 (69%), Female = 23 (31%)	60.0 (11.0)	Sodium and fluid restriction (meals)	0.8 g sodium and 0.8 mL fluid daily restriction in hospital 7 days or until discharge	*Weight loss and clinical stability (CCS)*, thirst, hospital readmissions	Aggressive sodium and fluid restriction has no effect on weight loss or clinical stability at 3 days and is associated with a significant increase in thirst	11
Rifai et al., 2015 [48]	2013	Israel	Middle East and North Africa	HIC	48; Male = 29 (60%), Female = 19 (40%)	62.0 (11.5)	DASH diet (information)	Provided with DASH eating plan guidebook and DASH shopping list, education from dietitian, 3 months	*Endothelial function*, MLHFQ, 6 min walk test, BNP	DASH diet group had better exercise capacity and quality of life scores at 3 months in patients with HF	9
Donner Alves et al., 2012 [49]	Not stated	Brazil	Latin America and the Caribbean	UMIC	46; Male = 32 (70%), Female = 14 (30%)	58.0 (10.0)	Global nutritional education (information)	Orientation on diet in HF, goal setting, and follow-up intensification as needed at one month	MLHFQ, weight loss, urinary sodium, dietary recall, nutrition knowledge	The nutritional education intervention improved nutritional knowledge, caloric, fat and sodium intake in outpatients with heart failure, but not quality of life or anthropometric parameters.	8
Hummel et al., 2018 [50]	Not stated	United States	North America	HIC	66; Male = 46 (70%), Female = 20 (30%)	71.0 (8.0)	Sodium-restricted DASH diet (meals)	Delivered meals tailored to DASH specifications for 12 weeks	*KCCQ*, BNP, hospital readmissions, length of stay, all-cause death	Home-delivered DASH meals after HF hospitalization appeared overall safe and had directionally favorable effects on HF clinical status and 30-day readmissions	10

Abbreviations: 6MWT (6 min walk test); BNP (Brain Natriuretic Peptide); CSS (Clinical Summary Score); DASH (Dietary Approaches to Stop Hypertension); ED (Emergency Department); HF (Heart Failure); HFpEF (Heart Failure with Preserved ejection Fraction); HIC (High-Income Country); KCCQ (Kansas City Cardiomyopathy Questionnaire); Kaiser Permanente Northern California (KPNC); SD (Standard Deviation); LVEDVI (Left Ventricular End-Diastolic Index); LVEF (Left Ventricular Ejection Fraction); LVESVI (Left Ventricular End-Systolic Volume Index); MLHFQ (Minnesota Living with Heart Failure Questionnaire); NT-proBNP (N-terminal pro-B-type natriuretic peptide); NYHA (New York Heart Association); QoL (Quality of Life); TMAO (trimethylamine N-oxide); UMIC (Upper Middle-Income Country). * Median (IQR). Mean unavailable. Risk of Bias was assessed using the Joanna Briggs Institute critical appraisal tool for Randomized Controlled Trials. The 0–13 score is provided as a summary sum of items scored as ‘Yes’. Details on individual question scores are provided in Supplementary Material Table S2.

## Data Availability

All data generated or analyzed during this study are included in this published article [and its Supplementary Information Files].

## Supplementary Materials

The following supporting information can be downloaded at https://www.mdpi.com/article/10.3390/jcm15218430/s1: Table S1: Database Search Strategies; Table S2: Validity Assessment with the JBI Critical Appraisal Checklist for Randomized Controlled Trials.

## Abbreviations

DASH	Dietary Approaches to Stop Hypertension
JBI	Joanna Briggs Institute
KCCQ	Kansas City Cardiomyopathy Questionnaire
NYHA	New York Heart Association
OR	Odds Ratio
PROSPERO	International Prospective Register of Systematic Reviews
RCT	Randomized Controlled Trial
