# Supplementary material for: Global Perspective on the Outcomes of Dietary Pattern and Nutrient-Based Interventions for Heart Failure: A Systematic Review of Randomized Controlled Trials
Source: J Clin Med. 2026 Oct 29;15(21):8430. doi: 10.3390/jcm15218430 (PMC13763981; doi:10.3390/jcm15218430)
Supplement: Supplementary file 1 [file jcm-15-08430-s001.zip › jcm-4614861-supplementary.pdf]

SUPPLEMENTARY MATERIALS

Table S1. Database Search Strategies

Database	Search Strategy	Results
Medline	1 nutrition therapy/ or diet therapy/ or diet, diabetic/ or diet, carbohydrate-restricted/ or exp diet, ketogenic/ or diet, fat-restricted/ or diet, gluten-free/ or diet, mediterranean/ or diet, paleolithic/ or diet, protein-restricted/ or diet, reducing/ or diet, sodium-restricted/ or exp diet, vegetarian/ or dietary approaches to stop hypertension/<br>2 diet/ or exp fasting/ or diet, healthy/ or diet*.mp. or nutrition.mp. [mp=title, book title, abstract, original title, name of substance word, subject heading word, floating sub-heading word, keyword heading word, organism supplementary concept word, protocol supplementary concept word, rare disease supplementary concept word, unique identifier, synonyms, population supplementary concept word, anatomy supplementary concept word]<br>3 heart failure/ or heart failure, diastolic/ or heart failure, systolic/<br>4 heart fail*.mp.<br>5 3 or 4<br>6 ((randomized controlled trial or controlled clinical trial).pt. or (Randomized or placebo or randomly or trial or groups).ab.) not (exp animals/ not exp humans/)<br>7 1 or 2<br>8 5 and 6 and 7<br>9 limit 8 to (yr="2012 -Current" and english)	1 59624<br>2 1137275<br>3 151859<br>4 266745<br>5 266745<br>6 3300029<br>7 1137275<br>8 1344<br>9 896
Cochrane Central Register of Controlled Trials	1 nutrition therapy/ or diet therapy/ or diet, diabetic/ or diet, carbohydrate-restricted/ or exp diet, ketogenic/ or diet, fat-restricted/ or diet, gluten-free/ or diet, mediterranean/ or diet, paleolithic/ or diet, protein-restricted/ or diet, reducing/ or diet, sodium-restricted/ or exp diet, vegetarian/ or dietary approaches to stop hypertension/<br>2 diet/ or exp fasting/ or diet, healthy/ or diet*.mp. or nutrition.mp. [mp=title, original title, abstract, floating sub-heading word, mesh headings, heading words, keyword]<br>3 heart failure/ or heart failure, diastolic/ or heart failure, systolic/<br>4 heart fail*.mp.	1 7496<br>2 137321<br>3 14379<br>4 37280

	5 3 or 4<br>6 ((randomized controlled trial or controlled clinical trial).pt. or (Randomized or placebo or randomly or trial or groups).ab.) not (exp animals/ not exp humans/)<br>7 1 or 2<br>8 5 and 6 and 7<br>9 limit 8 to (yr="2012 -Current" and english)	5 37280<br>6 1330967<br>7 137321<br>8 1159<br>9 850
PsycINFO	1 (MH "Diet Therapy+") OR nutrition therapy<br>2 (MH "Diet+") OR diet, healthy OR diet* OR nutrition<br>3 (MH "Heart Failure") OR heart failure, diastolic OR heart failure, systolic\<br>4 heart fail*<br>5 S3 OR S4<br>6 (MH (randomized controlled trials OR double-blind studies OR single-blind studies OR random assignment OR pretest-posttest design OR cluster sample) OR TI (randomised OR randomized) OR AB random* OR TI trial OR ((MH (sample size) AND AB (assigned OR allocated OR control))) OR MH (placebos OR crossover design OR comparative studies) OR AB ((control W5 group) OR (cluster W3 RCT) OR PT (randomized controlled trial))) NOT ((MH animals+ OR MH (animal studies) OR TI (animal model*)) NOT MH (human))<br>7 S1 OR S2<br>8 S5 AND S6 AND S7<br>9 S5 AND S6 AND S7 Limiters - Publication Year: 2012-2024; Language: English	1 271<br>2 92611<br>3 71<br>4 4868<br>5 4868<br>6 299842<br>7 92611<br>8 45<br>9 32
CINAHL	1 (MH "Diet Therapy+") OR nutrition therapy<br>2 (MH "Diet+") OR diet, healthy OR diet* OR nutrition<br>3 (MH "Heart Failure") OR heart failure, diastolic OR heart failure, systolic\<br>4 heart fail*<br>5 S3 OR S4<br>6 (MH (randomized controlled trials OR double-blind studies OR single-blind studies OR random assignment OR pretest-posttest design OR cluster sample) OR TI (randomised OR randomized) OR AB random* OR TI trial OR ((MH (sample size) AND AB (assigned OR allocated OR control))) OR MH (placebos OR crossover design OR comparative studies) OR AB ((control W5 group) OR (cluster W3 RCT) OR PT (randomized controlled trial))) NOT ((MH	1 39803<br>2 373719<br>3 50902<br>4 80768<br>5 80768<br>6 1000025

	animals+ OR MH (animal studies) OR TI (animal model*)) NOT MH (human))<br>7 S1 OR S2<br>8 S5 AND S6 AND S7<br>9 S5 AND S6 AND S7 Limiters - Publication Year: 2012-2024; Language: English	 <br>7 373719<br>8 534<br>9 371
Web of Science	1 ((TS=(nutrition therapy OR diet therapy OR diet, diabetic OR diet, carbohydrate-restricted OR diet, ketogenic OR diet, fat-restricted OR diet, gluten-free OR diet, mediterranean OR diet, paleolithic OR diet, protein-restricted OR diet, reducing OR (diet, sodium-restricted OR diet, vegetarian OR dietary approaches to stop hypertension))) OR TS=(diet OR fasting OR diet, healthy OR diet* OR nutrition))<br>2 (TS=(heart failure OR heart failure, diastolic OR heart failure, systolic)) OR TS=(heart fail*)<br>3 TS=(randomised OR randomized OR randomisation OR randomization OR placebo* OR (random* AND (allocat* OR assign*)) OR (blind* AND (single OR double OR treble OR triple))) NOT TS=(animal or animals or pisces or fish or fishes or catfish or catfishes or sheatfish or silurus or arius or heteropneustes or clarias or gariepinus or fathead minnow or fathead minnows or pimephales or promelas or cichlidae or trout or trouts or char or chars or salvelinus or salmo or oncorhynchus or guppy or guppies or millionfish or poecilia or goldfish or goldfishes or carassius or auratus or mullet or mullets or mugil or curema or shark or sharks or cod or cods or gadus or morhua or carp or carps or cyprinus or carpio or killifish or eel or eels or anguilla or zander or sander or lucioperca or stizostedion or turbot or turbots or psetta or flatfish or flatfishes or plaice or pleuronectes or platessa or tilapia or tilapias or oreochromis or sarotherodon or common sole or dover sole or solea or zebrafish or zebrafishes or danio or rerio or seabass or dicentrarchus or labrax or morone or lamprey or lampreys or petromyzon or pumpkinseed or pumpkinseeds or lepomis or gibbosus or herring or clupea or harengus or amphibia or amphibian or amphibians or anura or salientia or frog or frogs or rana or toad or toads or bufo or xenopus or laevis or bombina or epidalea or calamita or salamander or salamanders or newt or newts or triturus or reptilia or reptile or reptiles or bearded dragon or pogona or vitticeps or iguana or iguanas or lizard or lizards or anguis fragilis or turtle or turtles or snakes or snake	1 2399004<br>2 370594<br>3 1342866

	or aves or bird or birds or quail or quails or coturnix or bobwhite or colinus or virginianus or poultry or poultries or fowl or fowls or chicken or chickens or gallus or zebra finch or taeniopygia or guttata or canary or canaries or serinus or canaria or parakeet or parakeets or grasskeet or parrot or parrots or psittacine or psittacines or shelduck or tadorna or goose or geese or branta or leucopsis or woodlark or lullula or flycatcher or ficedula or hypoleuca or dove or doves or geopelia or cuneata or duck or ducks or greylag or graylag or anser or harrier or circus pygargus or red knot or great knot or calidris or canutus or godwit or limosa or lapponica or meleagris or gallopavo or jackdaw or corvus or monedula or ruff or philomachus or pugnax or lapwing or peewit or plover or vanellus or swan or cygnus or columbianus or bewickii or gull or chroicocephalus or ridibundus or albifrons or great tit or parus or aythya or fuligula or streptopelia or risoria or spoonbill or platalea or leucorodia or blackbird or turdus or merula or blue tit or cyanistes or pigeon or pigeons or columba or pintail or anas or starling or sturnus or owl or athene noctua or pochard or ferina or cockatiel or nymphicus or hollandicus or skylark or alauda or tern or sterna or teal or crecca or oystercatcher or haematopus or ostralegus or shrew or shrews or sorex or araneus or crocidura or russula or european mole or talpa or chiroptera or bat or bats or eptesicus or serotinus or myotis or dasycneme or daubentonii or pipistrelle or pipistrellus or cat or cats or felis or catus or feline or dog or dogs or canis or canine or canines or otter or otters or lutra or badger or badgers or meles or fitchew or fitch or foumart or foulmart or ferrets or ferret or polecat or polecats or mustela or putorius or weasel or weasels or fox or foxes or vulpes or common seal or phoca or vitulina or grey seal or halichoerus or horse or horses or equus or equine or equidae or donkey or donkeys or mule or mules or pig or pigs or swine or swines or hog or hogs or boar or boars or porcine or piglet or piglets or sus or scrofa or llama or llamas or lama or glama or deer or deers or cervus or elaphus or cow or cows or bos taurus or bos indicus or bovine or bull or bulls or cattle or bison or bisons or sheep or sheeps or ovis aries or ovine or lamb or lambs or mouflon or mouflons or goat or goats or capra or caprine or chamois or rupicapra or leporidae or lagomorpha or lagomorph or rabbit or rabbits or oryctolagus or cuniculus or laprine or hares or lepus or rodentia or rodent or rodents or murinae or	

	mouse or mice or mus or musculus or murine or woodmouse or apodemus or rat or rats or rattus or norvegicus or guinea pig or guinea pigs or cavia or porcellus or hamster or hamsters or mesocricetus or cricetulus or cricetus or gerbil or gerbils or jird or jirds or meriones or unguiculatus or jerboa or jerboas or jaculus or chinchilla or chinchillas or beaver or beavers or castor fiber or castor canadensis or sciuridae or squirrel or squirrels or sciurus or chipmunk or chipmunks or marmot or marmots or marmota or suslik or susliks or spermophilus or cynomys or cottonrat or cottonrats or sigmodon or vole or voles or microtus or myodes or glareolus or primate or primates or prosimian or prosimians or lemur or lemurs or lemuridae or loris or bush baby or bush babies or bushbaby or bushbabies or galago or galagos or anthropoidea or anthropoids or simian or simians or monkey or monkeys or marmoset or marmosets or callithrix or cebuella or tamarin or tamarins or saguinus or leontopithecus or squirrel monkey or squirrel monkeys or saimiri or night monkey or night monkeys or owl monkey or owl monkeys or douroucoulis or aotus or spider monkey or spider monkeys or ateles or baboon or baboons or papio or rhesus monkey or macaque or macaca or mulatta or cynomolgus or fascicularis or green monkey or green monkeys or chlorocebus or vervet or vervets or pygerythrus or hominoidea or ape or apes or hylobatidae or gibbon or gibbons or siamang or siamangs or nomascus or symphalangus or hominidae or orangutan or orangutans or pongo or chimpanzee or chimpanzees or pan troglodytes or bonobo or bonobos or pan paniscus or gorilla or gorillas or troglodytes)	
	4 #1 AND #2 AND #3	4 1439
	5 #4 Timespan: 2012-01-01 to 2024-04-30	5 948

Note: Numbers correspond to initial search between January 2012 and April 2024. This was supplemented by a search update using the same MEDLINE (Ovid) approach for literature published between April 2024 and October 2026 in which 370 new results were identified and 5 new studies added to final analysis.

Table S2. Validity Assessment with the JBI Critical Appraisal Checklist for Randomized Controlled Trials

Study	Q1	Q2	Q3	Q4	Q5	Q6	Q7	Q8	Q9	Q10	Q11	Q12	Q13	Total
	Internal Validity										Statistical Conclusion Validity			
Eberly et al., 2026	Y	U	U	N	U	Y	Y	N	Y	Y	Y	Y	Y	8
Pandey et al., 2026	Y	U	Y	N	U	U	N	N	Y	Y	Y	Y	Y	7
Compher et al., 2025	Y	Y	Y	N	N	Y	Y	N	Y	Y	N	Y	Y	9
Li et al., 2025	Y	U	Y	N	N	U	Y	N	Y	Y	Y	N	Y	7
Ortiz-Cortés et al., 2025	Y	Y	Y	N	N	Y	Y	Y	Y	Y	Y	N	Y	10
Zhou et al., 2023	Y	U	Y	N	N	U	Y	U	Y	Y	Y	N	Y	7
Ivey-Miranda et al., 2023	Y	Y	Y	Y	U	Y	Y	Y	Y	Y	Y	Y	Y	12
Go et al., 2022	Y	U	Y	N	U	Y	Y	Y	Y	Y	Y	Y	Y	10
Ezekowitz et al., 2022	Y	N	Y	N	N	Y	Y	Y	Y	Y	Y	Y	Y	10
Evangelista et al., 2021	U	U	Y	U	N	U	Y	Y	Y	Y	Y	Y	Y	8
Kalogeropoulos at al., 2020	U	U	Y	Y	N	Y	Y	Y	Y	Y	Y	Y	Y	10
Fabricio et al., 2019	Y	Y	Y	U	Y	Y	Y	Y	Y	Y	Y	Y	Y	12
Machado d'Almeida et al., 2018	Y	Y	Y	N	N	Y	Y	Y	Y	Y	Y	Y	Y	11
Gonzalez-Islas et al., 2017	Y	N	Y	N	N	Y	Y	Y	Y	Y	Y	Y	Y	10
Bonilla-Palomas et al., 2016	Y	U	Y	N	N	U	Y	Y	Y	Y	Y	Y	Y	9
Colin-Ramirez et al., 2015	Y	U	Y	N	N	Y	Y	Y	Y	Y	Y	Y	Y	10
Philipson et al., 2013	Y	U	Y	N	N	Y	Y	Y	Y	Y	Y	Y	Y	10
Aliti et al., 2013	Y	Y	Y	U	N	Y	Y	Y	Y	Y	Y	Y	Y	11
Rifai et al., 2015	Y	U	Y	N	N	N	Y	Y	Y	Y	Y	Y	Y	9
Donner Alves et al., 2012	Y	U	N	N	N	U	Y	Y	Y	Y	Y	Y	Y	8
Hummel et al., 2018	Y	U	Y	N	U	Y	Y	Y	Y	Y	Y	Y	Y	10

Note: JBI (Joanna Briggs Institute); Answers: Yes, No, Unclear or Not/Applicable

<u>Q1-Q13: Questions 1 to 13</u>

1. Was true randomization used for assignment of participants to treatment groups?
2. Was allocation to treatment groups concealed?
3. Were treatment groups similar at the baseline?

4. Were participants blind to treatment assignment?
5. Were those delivering treatment blind to treatment assignment?
6. Were outcomes assessors blind to treatment assignment?
7. Were treatment groups treated identically other than the intervention of interest?
8. Was follow up complete and if not, were differences between groups in terms of their follow up adequately described and analyzed?
9. Were participants analyzed in the groups to which they were randomized?
10. Were outcomes measured in the same way for treatment groups?
11. Were outcomes measured in a reliable way?
12. Was appropriate statistical analysis used?
13. Was the trial design appropriate, and any deviations from the standard RCT design (individual randomization, parallel groups) accounted for in the conduct and analysis of the trial?

PRISMA 2020 Checklist

Section and Topic	Item #	Checklist item	Location where item is reported
**TITLE**			
Title	1	Identify the report as a systematic review.	1
**ABSTRACT**			
Abstract	2	See the PRISMA 2020 for Abstracts checklist.	1
**INTRODUCTION**			
Rationale	3	Describe the rationale for the review in the context of existing knowledge.	2 – 4
Objectives	4	Provide an explicit statement of the objective(s) or question(s) the review addresses.	4
**METHODS**			
Eligibility criteria	5	Specify the inclusion and exclusion criteria for the review and how studies were grouped for the syntheses.	4-5
Information sources	6	Specify all databases, registers, websites, organisations, reference lists and other sources searched or consulted to identify studies. Specify the date when each source was last searched or consulted.	5
Search strategy	7	Present the full search strategies for all databases, registers and websites, including any filters and limits used.	7
Selection process	8	Specify the methods used to decide whether a study met the inclusion criteria of the review, including how many reviewers screened each record and each report retrieved, whether they worked independently, and if applicable, details of automation tools used in the process.	4-5
Data collection process	9	Specify the methods used to collect data from reports, including how many reviewers collected data from each report, whether they worked independently, any processes for obtaining or confirming data from study investigators, and if applicable, details of automation tools used in the process.	5-6
Data items	10a	List and define all outcomes for which data were sought. Specify whether all results that were compatible with each outcome domain in each study were sought (e.g. for all measures, time points, analyses), and if not, the methods used to decide which results to collect.	5-6
	10b	List and define all other variables for which data were sought (e.g. participant and intervention characteristics, funding sources). Describe any assumptions made about any missing or unclear information.	5-12
Study risk of bias assessment	11	Specify the methods used to assess risk of bias in the included studies, including details of the tool(s) used, how many reviewers assessed each study and whether they worked independently, and if applicable, details of automation tools used in the process.	11-12
Effect measures	12	Specify for each outcome the effect measure(s) (e.g. risk ratio, mean difference) used in the synthesis or presentation of results.	12
Synthesis methods	13a	Describe the processes used to decide which studies were eligible for each synthesis (e.g. tabulating the study intervention characteristics and comparing against the planned groups for each synthesis (item #5)).	11-12
	13b	Describe any methods required to prepare the data for presentation or synthesis, such as handling of missing summary statistics, or data conversions.	NA
	13c	Describe any methods used to tabulate or visually display results of individual studies and syntheses.	5-12
	13d	Describe any methods used to synthesize results and provide a rationale for the choice(s). If meta-analysis was performed, describe the model(s), method(s) to identify the presence and extent of statistical heterogeneity, and software package(s) used.	11-12
	13e	Describe any methods used to explore possible causes of heterogeneity among study results (e.g. subgroup analysis, meta-regression).	NA
	13f	Describe any sensitivity analyses conducted to assess robustness of the synthesized results.	NA
Reporting bias assessment	14	Describe any methods used to assess risk of bias due to missing results in a synthesis (arising from reporting biases).	12
Certainty assessment	15	Describe any methods used to assess certainty (or confidence) in the body of evidence for an outcome.	12

PRISMA 2020 Checklist

Section and Topic	Item #	Checklist item	Location where item is reported
**RESULTS**			
Study selection	16a	Describe the results of the search and selection process, from the number of records identified in the search to the number of studies included in the review, ideally using a flow diagram.	12-15
	16b	Cite studies that might appear to meet the inclusion criteria, but which were excluded, and explain why they were excluded.	NA
Study characteristics	17	Cite each included study and present its characteristics.	10-11
Risk of bias in studies	18	Present assessments of risk of bias for each included study.	Table 1, Supplementary Table B
Results of individual studies	19	For all outcomes, present, for each study: (a) summary statistics for each group (where appropriate) and (b) an effect estimate and its precision (e.g. confidence/credible interval), ideally using structured tables or plots.	Table 1, pgs 15-18
Results of syntheses	20a	For each synthesis, briefly summarise the characteristics and risk of bias among contributing studies.	15-18
	20b	Present results of all statistical syntheses conducted. If meta-analysis was done, present for each the summary estimate and its precision (e.g. confidence/credible interval) and measures of statistical heterogeneity. If comparing groups, describe the direction of the effect.	NA
	20c	Present results of all investigations of possible causes of heterogeneity among study results.	NA
	20d	Present results of all sensitivity analyses conducted to assess the robustness of the synthesized results.	NA
Reporting biases	21	Present assessments of risk of bias due to missing results (arising from reporting biases) for each synthesis assessed.	Table 1, pgs 15-18
Certainty of evidence	22	Present assessments of certainty (or confidence) in the body of evidence for each outcome assessed.	15-18
**DISCUSSION**			
Discussion	23a	Provide a general interpretation of the results in the context of other evidence.	20-22
	23b	Discuss any limitations of the evidence included in the review.	22-23
	23c	Discuss any limitations of the review processes used.	22-23
	23d	Discuss implications of the results for practice, policy, and future research.	23-24
**OTHER INFORMATION**			
Registration and protocol	24a	Provide registration information for the review, including register name and registration number, or state that the review was not registered.	4
	24b	Indicate where the review protocol can be accessed, or state that a protocol was not prepared.	4
	24c	Describe and explain any amendments to information provided at registration or in the protocol.	NA
Support	25	Describe sources of financial or non-financial support for the review, and the role of the funders or sponsors in the review.	25
Competing interests	26	Declare any competing interests of review authors.	25
Availability of data, code and other materials	27	Report which of the following are publicly available and where they can be found: template data collection forms; data extracted from included studies; data used for all analyses; analytic code; any other materials used in the review.	NA

PRISMA 2020 Checklist

From: Page MJ, McKenzie JE, Bossuyt PM, Boutron I, Hoffmann TC, Mulrow CD, et al. The PRISMA 2020 statement: an updated guideline for reporting systematic reviews. BMJ 2021;372:n71. doi: 10.1136/bmj.n71
